# Tumour aneuploidy, prognostic parameters and survival in primary breast cancer.

**DOI:** 10.1038/bjc.1987.88

**Published:** 1987-04

**Authors:** A. A. Owainati, R. A. Robins, C. Hinton, I. O. Ellis, C. S. Dowle, B. Ferry, C. W. Elston, R. W. Blamey, R. W. Baldwin

## Abstract

Cellular DNA content of primary tumours from 280 patients with operable breast cancer was determined by flow cytometry using nuclei from paraffin sections stained with DAPI, and 199 of these patients were followed for 8-13 years after surgery. Tumours from 67 patients have also been analyzed for their DNA content using single cell suspensions from fresh tumour tissue stained with mithramycin and ethidium bromide, and the results compared with those obtained from paraffin blocks of the same tumours. Overall 60% of the tumours contained cells with abnormal DNA content (DNA-aneuploid populations). Survival and disease free interval were not significantly different in patients with DNA-diploid and DNA-aneuploid tumours when analysed by Mantel's life table method. There was however, an early advantage for patients with DNA-diploid tumours: during the first 30 months after surgery DNA-aneuploidy was associated with higher rate of recurrence and shorter survival. DNA-aneuploidy was strongly related to histological grade. Thus 11/49 (22%) grade I, 60/102 (59%) grade II, and 96/129 (74%) grade III tumours were DNA-aneuploid. Although there was no significant difference in survival of patients with DNA-diploid and DNA-aneuploid tumours overall, there appears to be an unexpected association between DNA-aneuploidy and better survival in grade II patients (P less than 0.01); a similar trend was observed for grade I patients. Although the proportion of DNA-aneuploid tumours was similar in oestrogen receptor positive and negative tumours, DNA-aneuploidy was associated with lower levels of oestrogen receptors in comparison to DNA-diploid tumours. Comparison between the modal DNA values of fresh and paraffin embedded samples showed high rate of comparability (64/67, P less than 0.0001).


*Br. J. Cancer* (1987), **55**, 449–454 © The Macmillan Press Ltd., 1987

### Tumour aneuploidy, prognostic parameters and survival in primary breast cancer

A.A.R. Owainati[1], R.A. Robins[1], C. Hinton[2], I.O. Ellis[3], C.S. Dowle[2], B. Ferry[1], C.W. Elston[3], R.W. Blamey[2] & R.W. Baldwin[1]

[1]*Cancer Research Campaign Laboratories,* [2]*Department of Surgery and* [3]*Department of histopathology, University of Nottingham, Nottingham NG7 2RD, UK.*

**Summary** Cellular DNA content of primary tumours from 280 patients with operable breast cancer was determined by flow cytometry using nuclei from paraffin sections stained with DAPI, and 199 of these patients were followed for 8–13 years after surgery. Tumours from 67 patients have also been analyzed for their DNA content using single cell suspensions from fresh tumour tissue stained with mithramycin and ethidium bromide, and the results compared with those obtained from paraffin blocks of the same tumours.

Overall 60% of the tumours contained cells with abnormal DNA content (DNA-aneuploid populations). Survival and disease free interval were not significantly different in patients with DNA-diploid and DNA-aneuploid tumours when analysed by Mantel's life table method. There was however, an early advantage for patients with DNA-diploid tumours: during the first 30 months after surgery DNA-aneuploidy was associated with higher rate of recurrence and shorter survival. DNA-aneuploidy was strongly related to histological grade. Thus 11/49 (22%) grade I, 60/102 (59%) grade II, and 96/129 (74%) grade III tumours were DNA-aneuploid. Although there was no significant difference in survival of patients with DNA-diploid and DNA-aneuploid tumours overall, there appears to be an unexpected association between DNA-aneuploidy and better survival in grade II patients ($P<0.01$); a similar trend was observed for grade I patients. Although the proportion of DNA-aneuploid tumours was similar in oestrogen receptor positive and negative tumours, DNA-aneuploidy was associated with lower levels of oestrogen receptors in comparison to DNA-diploid tumours. Comparison between the modal DNA values of fresh and paraffin embedded samples showed high rate of comparability (64/67, $P<0.0001$).

The DNA content of the cells in a tumour is becoming a frequently measured tumour characteristic. It is a feature which is variable between tumours of a given site (Atkin & Richards, 1956), and as it involves evaluation of a degree of abnormality in the genetically heritable components of the cell, might be expected to relate to the biological behaviour of the tumour. However, the significance of this putative relationship between abnormal DNA content and biological behaviour with respect to the clinical outcome with different types of tumour is still not fully established.

Many studies have shown significant correlations in breast cancer between cellular DNA content and other clinical and pathological features of the tumour suggestive of poor clinical outcome for patients with DNA-aneuploid tumours (see e.g. Olszewski *et al.*, 1981; Fossa *et al.*, 1982; Raber *et al.*, 1982; Taylor *et al.*, 1983; Auer *et al.*, 1984; Hedley *et al.*, 1985; Thorud *et al.*, 1986). A few studies have shown a significant difference in the survival of patients with diploid and aneuploid tumours (Atkin 1972; Atkin & Kay 1979; Auer *et al.*, 1980; 1984) and in the disease-free interval (Auer *et al.*, 1984; Ewers *et al.*, 1984; Hedley *et al.*, 1984; 1985). However, studies in other centres have not found such correlations or the correlations did not reach statistical significance (Raber *et al.*, 1982; Taylor *et al.*, 1983; Coulson *et al.*, 1984; Stuart-Harris *et al.*, 1985).

Therefore, the significance of the presence of a cell population with abnormal DNA content in breast cancer has not been fully resolved. More work is needed to define the influence of this tumour characteristic on patient survival, and its relationship to other features and properties of the tumour of known prognostic significance.

This paper describes our investigations into the determination of cellular DNA content in primary breast cancer, including comparison between techniques using fresh tumour tissue and nuclei derived from paraffin embedded tumours, and the prognostic significance of aneuploidy determined by these methods. A subsequent paper will explore in more detail the interrelationships between cellular DNA content and clinico-pathological features of the tumour, and how these are in turn related to clinical outcome in different subgroups of patients (Dowle *et al.*, 1987).

Correspondence: R.A. Robins.
Received 2 July 1986; and in revised form 10 November 1986.

### Materials and methods

### *Patients*

Tumour samples were obtained from patients with primary operable invasive breast cancer. These patients were from a consecutive series under the care of a single surgical team, treated by simple mastectomy. Triple lymph node biopsy was undertaken at mastectomy for histopathological staging: patients without lymph node involvement were stage A; only lower axillary involvement stage B; and upper axilla or internal mammary chain involvement stage C. No further treatment was given until recurrence, when patients were given endocrine or chemotherapy.

### *Analysis of fixed tissue*

Nuclei from 30 $\mu$m sections of paraffin embedded tumour tissue were analysed for nuclear DNA content. 5 $\mu$m sections were also obtained and examined histologically to confirm the presence of malignant cells in the material analysed. Essentially following the procedure of Hedley *et al.*, 1983, two sections were dewaxed in xylene, rehydrated in graded alcohols, and digested in pepsin (Sigma, 0.5% in saline pH 1.5) in a 37°C water bath with frequent vortexing. Nuclei isolated after centrifugation were stained with diaminido-phenyl indol (DAPI, Sigma) (1 $\mu g^{-1}$), and held on ice until analysed.

### *Analysis of fresh tumours*

Fresh tumour samples were obtained from 67 consecutive patients of the same series and put in sterile minimum essential medium (MEM) and kept at 4°C until analysed within 24 h of surgery. The tumour sample was chopped into

2–3 mm³ pieces and treated with 10 ml collagenase (0.5 mg ml$^{-1}$) (type I, Boehringer Mannheim) and 2–3 drops of 0.02% DNAase, to prevent cells clumping. The mixture was gently mixed with a magnetic stirrer for 20 min at 37°C. Dissociated nucleated cells were washed twice with Hanks' salt medium and $2 \times 10^5$ tumour cells in 0.1 ml were mixed with 0.1% Triton X100 for 1 min, to render the cell membrane permeable to the staining fluorochromes, and then treated with 0.5 ml of ethidium bromide (final concentration 15 $\mu$g ml$^{-1}$) (Sigma) and mithramycin (final concentration 37.4 $\mu$g ml$^{-1}$) (Sigma). After 30 min incubation at 4°C the cells were analysed by flow cytometry.

*Flow cytometry*

Flow analysis was done using FACS IV (Becton Dickinson FACS system). DAPI fluorescence was excited using 40 mW UV light from an argon laser and collected via a 488 band pass filter with a 10 nM bandwidth. The mean coefficient of variation of the G0/G1 peak was 6.6% (range 4.9–8.7). Mithramycin fluorescence was excited using 120 mW light at 457 nM from an argon laser and fluorescence collected via 520 long pass filters.

DNA index (DI) was determined according to a recently suggested convention on nomenclature for DNA cytometry (Hiddemann *et al.*, 1984). Thus DI was defined as the ratio of the mode of the relative DNA content of the G0/G1 cells of the sample divided by the mode of the relative DNA measurement of the diploid G0/G1 reference cells. Cells with a normal diploid karyotype have, by definition a DI of 1.0 (Figure 1a). A DNA histogram was said to contain a DNA-aneuploid population when at least two separate G0/G1 peaks were demonstrable (Figure 1c) and DI greater or lower than 1.0. Where the putative aneuploid peak was at or near the G2 position (DI 1.9–2.1), an arbitary cut-off point was used, so that peaks containing more than 15% of the cells in the distribution were defined as DNA-aneuploid.

Normal human lymphocytes were stained by the same procedure with each batch of fresh tumours analysed and run before the samples to calibrate the instrument for the position of G0/G1 normal DNA-diploid peak. Each tumour sample is a suspension of normal and malignant cells and the normal cells in the tumour sample, identified by the position of their peak on the same channel of the prerun normal lymphocytes, were used as internal diploid standard for DI calculation.

For the paraffin embedded samples no internal standard, apart from the normal cells within the tumour sample, was used as neither chicken RBC nor human peripheral lymphocytes give consistent ratios to the diploid peak when added to the pepsin digests of embedded tissue (Hedley *et al.*, 1983). Therefore, the first peak in the histogram was considered as G0/G1 normal diploid standard.

For the purposes of comparison of data from fresh and paraffin sections, the DI for the tumours found to be hypodiploid in the analysis of fresh tissue were recalculated assuming the first peak to be diploid, as is done for the paraffin sections.

*Histological grading*

Histological assessment was done by a modification of the method of Bloom and Richardson, 1957 (Elston *et al.*, 1982). Tumours are graded on a scale from grade I (well differentiated), to grade III (poorly differentiated), using a scoring system which takes into account tubule formation, nuclear pleomorphism, and mitotic activity.

*Oestrogen receptors assay*

The assays have been performed by Tenovus institute (Cardiff) by the dextran coated charcoal method (Nicholson *et al.*, 1981). Oestrogen receptor was considered positive when a value of greater than 5 fmol mg$^{-1}$ cytosol was found.

*Prognostic grouping of patients*

Prognostic index was calculated according to Haybittle *et al.*, 1982. This index takes account of the size, lymph node stage, and histological grade of tumours to allocate patients into one of three prognostic groups. The groups are: (1) a good prognosis group whose survival is similar to that of a non-cancerous aged matched population, (2) a moderate prognosis group, and (3) a poor prognosis group whose survival at 5 years is <20%.

*Statistical evaluation of survival and disease free interval (DFI)*

Mantel's life table analysis was used to compare survival and DFI between different patient groups.

### Results

*Distribution of DI values*

Overall, 60% (167/280) of the primary breast tumours examined contained DNA-aneuploid cell populations. The distribution of DI values is shown in Figure 2. The aneuploid DNA indices show a relatively narrow distribution, with the majority of values between 1.6 and 2.0.

*Comparison fixed and fresh samples*

Results of analysis of paraffin sections (Figure 1a, c) with those obtained by conventional analysis of fresh tumour specimens from the same patients (Figure 1b, d), showed comparable DNA histograms, and 64/67 showed comparable DI (Figure 3). Applying Student's *t*-test to the differences between the DI values of the two methods showed a $t_0$-value of 1.5144 (not significant). The expected difference between

**Figure 1** DNA histograms from two primary breast carcinomas: paraffin section nuclei (a, c) and fresh tumour tissue (b, d) of DNA-diploid (a, b) and DNA-aneuploid (c, d) tumours.

**Figure 2** Distribution of DNA index (DI) values for 280 primary breast carcinomas.

**Figure 3** Comparison of DNA index (DI) for 67 tumours determined by analysis of fresh tumour tissue and paraffin section nuclei.

the DI values obtained by the two methods, at 95% confidence, lies in the range +0.07 and −0.009.

*DNA ploidy and recurrence*

The results of long term follow up of 199 patients with primary operable breast cancer showed that at the end of 8 years there was no difference in the rate of recurrence of DNA-diploid and DNA-aneuploid tumours (Figure 4). DNA-aneuploid tumours, however, were more likely to recur in the first 2 years following the removal of the primary tumour, than DNA-diploid tumours (Table I), although there was no difference in the overall disease free interval

**Figure 4** Disease free survival of patients with DNA-diploid (▲) and DNA-aneuploid (●) breast carcinomas, analysed by Mantel's life table method.

**Table 1** Correlation of DI to recurrence in 2 years and after 2 years

| | *<2 years* | *> 2 years* |
|---|---|---|
| DNA-diploid | 22(33%) | 28(52%) |
| DNA-aneuploid | 44(66%) | 26(48%) |

$\chi^2 = 4.19$; $P < 0.05$.

(Figure 4). Also, there was no difference in survival after recurrence between patients with DNA-diploid and DNA-aneuploid tumours.

*DNA ploidy and survival*

Overall survival of the patients was not significantly different between those with DNA-diploid and DNA-aneuploid tumours after 8–13 years follow up when tested by Mantel's life table analysis (Figure 5). There is an early difference in the survival of the two groups of patients which is statistically significant at 30 months ($\chi^2$ 3.768, $P < 0.05$), which agrees with the observations of the others on short follow up (Hedley *et al.*, 1985). However, by 3 years after surgery, there is little difference in survival. Tetraploid tumours have been shown to have a similar disease course to

**Figure 5** Survival of patients with DNA-diploid (▲) and DNA-aneuploid (●) breast carcinoma, analysed by Mantel's life table method.

DNA-diploid tumours in several studies (Atkin, 1972; Auer *et al.*, 1980; 1984). When tetraploid tumours were excluded from our analysis, there was still no significant difference in the survival of patients with DNA-aneuploid and DNA-diploid tumours.

### *Relationship between DNA ploidy and histological grade*

DNA ploidy was strongly related to the histological grade of differentiation of the tumours ($P<0.0001$; Table II). Thus well differentiated tumours were predominantly DNA-diploid (78%) whilst the poorly differentiated tumours were mostly DNA-aneuploid (74%). In view of the strong prognostic significance of histological grade (Elston *et al.*, 1982), and the weakness of the relationship between ploidy and survival shown above, it is surprising that such a strong correlation is observed between ploidy and grade. Furthermore, detailed analysis shows that DNA-aneuploidy tends to be associated with better survival in patients with grade I and II tumours (Figure 6a, b). This relationship was significant with grade II ($P<0.01$). No difference in overall survival between patients with DNA-aneuploid and DNA-diploid tumours was observed in grade III patients (Figure 6c).

**Table II** Correlation between ploidy and histological grade

| | *Grade I* | *Grade II* | *Grade III* |
|---|---|---|---|
| DNA-diploid | 38(78%)* | 42(41%) | 33(26%) |
| DNA-aneuploid | 11(22%) | 60(59%) | 96(74%) |

$\chi^2=26.1036$; $P<0.0001$

*percentage of cases within each grade.

### *Relationship between DNA ploidy and ER level*

Oestrogen receptor status had been assessed in 252 of the cases studied by DNA analysis. The level of ER varied between 0–396 fmol mg$^{-1}$ cytosol. One hundred and twenty-one of the tumours (48%) had an ER level >5 fmol mg$^{-1}$ cytosol, conventionally defined as the ER+ group. The distribution of DNA-diploid and DNA-aneuploid tumours was not significantly different in the ER+ and ER− groups defined in this way. However, DNA-diploid tumours were associated with higher levels of ER, whereas DNA-aneuploid tumours were associated with lower levels (Table III). This correlation was statistically significant ($\chi^2$ 6.475, $P<0.05$).

### *Relationship between DNA ploidy and lymph node stage*

There was no statistically significant relationship between DNA ploidy and tumour metastasis to regional lymph nodes defined by histopathological examination of nodes sampled at the time of mastectomy (Table IV).

**Figure 6** Survival of patients with DNA-diploid (▲) and DNA-aneuploid (●) tumours according to histological grade: grade I (a.), grade II (b.) and grade III (c.).

**Table III** Correlation of DI with ER level

| | *ER value, fmol mg$^{-1}$ cytosol protein* | | | |
|---|---|---|---|---|
| | *<6* | *6–10* | *11–50* | *>50* |
| DNA-diploid | 42(42%)* | 2(2%) | 25(25%) | 31(31%) |
| DNA-aneuploid | 79(52%) | 9(6%) | 24(16%) | 40(26%) |

$\chi^2=6.475$; $P<0.5$.
*percentage of cases within each ploidy type.

**Table IV** Correlation of DI to stage

| | *A* | *B* | *C* |
|---|---|---|---|
| DNA-diploid | 66(44%) | 29(35%) | 18(38%) |
| DNA-aneuploid | 84(56%) | 54(65%) | 29(62%) |

$\chi^2=1.921$, ns.

### *Relationship between DNA ploidy and prognostic grouping*

Grouping the patients according to the prognostically significant pathological features (Nottingham prognostic index), showed significant difference in the distribution of

**Table V** Correlation of DI to prognostic groups

| | *Good* | *Moderate* | *Poor* |
|---|---|---|---|
| DNA-diploid | 27 | 40 | 13 |
| DNA-aneuploid | 18 | 69 | 32 |

$\chi^2 = 9.499$; $P < 0.005$.

DNA-diploid and DNA-aneuploid tumours within these groups ($P<0.001$) (Table V). DNA-diploid tumours were more likely to be associated with good or moderate prognosis (49 and 52/112, respectively), while DNA-aneuploid tumours were more likely to be associated with moderate or poor prognosis (91 & 37/166, respectively).

### Discussion

In this study we found that DNA analysis of paraffin embedded tissue gave DNA index results that were in general comparable with those obtained by analysis of a fresh tissue sample, confirming the experience of Hedley *et al.*, 1983. There were two cases of disagreement where an aneuploid population was found from analysis of a paraffin section but not after dissociation of fresh tissue. It was suspected that selective loss of malignant cells during tissue dissociation might be a source of this type of disagreement between methods, although this seems to occur relatively infrequently. There was also an example of a tumour in which an aneuploid population found on analysis of fresh tissue was not found in the paraffin section material. This was presumably due to heterogeneity within the tumour, and further studies may be required to define the frequency with which this variation occurs.

The generally good agreement between methods enabled us to use archival material to look retrospectively at the relationship between abnormal DNA content and clinical course and survival of a consecutive series of patients with primary operable tumour who have undergone simple mastectomy and no further therapy until recurrence.

These data show that there is a complex relationship between tumour DNA ploidy and patient survival. Thus, DNA-aneuploidy correlates significantly with other features known to be associated with poor prognosis in breast cancer, including poor histological grade and lower levels of oestrogen receptors, as has been found by others (for e.g., Fossa *et al.*, 1982; Olszewski *et al.*, 1981; Raber *et al.*, 1982; Taylor *et al.*, 1983; Hedley *et al.*, 1984). We also show here a significant correlation to the Nottingham prognostic index, which takes into account tumour size, regional lymph node involvement, and histological grade. Although there was no significant relationship between DNA aneuploidy and stage, it is surprising that patients with DNA-aneuploid tumours do not suffer a significantly worse survival overall.

It should be pointed out that there is poorer survival of patients with DNA-aneuploid tumours in short term follow up, as judged by Chi square analysis of survival during the first 30 months after surgery. However, longer follow up fails to show any difference in the survival of patients with DNA-diploid and DNA-aneuploid tumours.

Disease free survival is also not significantly different between patients with DNA-diploid and DNA-aneuploid tumours, according to Mantel's life table analysis. DNA-aneuploid tumours tended to recur more quickly, in the first two years following removal of the primary tumour, in contrast to DNA-diploid tumours in which the recurrences were more evenly distributed through the follow-up period. This difference in the recurrence rate in the early years of follow up is in agreement with the studies of the others (Ewers *et al.*, 1984; Hedley *et al.*, 1984; 1985; Thorud *et al.*, 1986), and is consistent with the findings of others who take two different time points of follow up to reflect the disease free survival significance of DNA ploidy (Auer *et al.*, 1984). Survival after recurrence was not different whether the tumour was DNA-diploid or DNA-aneuploid, in agreement with the observation of Stuart-Harris *et al.*, (1985). Also, there was no apparent relationship between response to treatment and DNA-aneuploidy.

There appears to be some inconsistency between the results of studies using static cytometry (e.g. Auer *et al.*, 1984) and flow methods. This may reflect a difference in sensitivity, as flow methods may not detect small populations of aneuploid cells which might be selected for measurement in static cytometry. However, it is clear that there are many patients whose tumours contain frank, easily detected DNA-aneuploid populations, but whose prognosis is not poor in comparison with similar patients with DNA-diploid tumours.

Another complex aspect of the relationship between DNA-aneuploidy and survival is the interaction with histological grade. Thus there appears to be longer survival of patients with DNA-aneuploid tumours that are moderately differentiated (grade II), in comparison with DNA-diploid tumours of the same histological grades. A similar trend is observed for grade I patients, although there are few DNA-aneuploid tumours in this group, and the difference is not significant by life table analysis. The failure of a subgroup of patients with DNA-aneuploid breast tumours to do badly in comparison to their DNA-diploid counterparts is not without precedent. For example, in a small group of patients undergoing treatment for advanced disease, Stuart-Harris *et al.*, (1985) found a trend for patients with DNA-aneuploid tumours to survive better.

Further explanation is required for the paradox that although DNA-aneuploidy is strongly related to histological grade, and grade is strongly related to survival, DNA-aneuploidy is not predictive of poor survival. One possibility is that DNA-aneuploidy correlates with an element of grade that does not strongly relate to survival. To investigate this point, comparisons have been made between DNA-aneuploidy and objective measurements of nuclear features of some of the tumours in this study; the detail of these measurements will be reported separately (Ellis *et al.*, in preparation). DNA-diploid tumours were found to be associated with smaller size nuclei with limited variations in nuclear area and perimeter and almost all DNA-diploid tumours were associated with the lowest rate of mitosis, whereas DNA-aneuploid tumours were associated with larger nuclei with a wide range of variation in nuclear area and perimeter, and contained a wide range of mitotic activity. This suggests that low proliferative activity is found in DNA-diploid tumours, in contrast to DNA-aneuploid tumours where an extended range of proliferative activity was observed. When the patients were grouped into different survival groups according to these nuclear morphometric measurements and mitotic rate, the long survival groups were predominantly DNA-diploid. DNA-aneuploid tumours were distributed over a wide range of survival, which seems to obscure the survival advantage of DNA-diploid tumours.

These studies show that DI is a marker which relates to prognostically significant features in breast cancer, and DNA-diploidy may in particular be associated with low proliferative activity. However, DI is not useful as a single marker for prediction of survival. More detailed analysis will be required to assess the possibility of using DI as a factor, with the other prognostically significant parameters, contributing to prognosis in primary breast cancer.

The authors wish to express their gratitude to Mr J. Lawry and Mr O.F. Roberts for their skillful technical assistance, and Mrs M. Trevers for typing the manuscript. This work was supported by a grant from the Cancer Research Campaign.

### References

ATKIN, N.B., & RICHARDS, B.M. (1956). DNA in human tumours as measured by microspectrophotometry of Feulgen stain: a comparison of tumours arising at different sites. *Br. J. Cancer*, **13**, 769.

ATKIN, N.B. (1972). Modal DNA value and survival in carcinoma of the breast. *Br. Med. J.*, **1**, 271.

ATKIN, N.B. & KAY, R. (1979). Prognostic significance of modal DNA and other factors in malignant tumours, based on 1465 cases. *Br. J. Cancer*, **40**, 210.

AUER, G., CASPERSSON, T. & WALLGREN, A. (1980). DNA content and survival in mammary carcinoma. *Anal. Quant. Cytology*, **2**, 161.

AUER, G., ERIKSSON, E., AZAVEDO, E., CASPERSSON, T. & WALLGREN, A. (1984). Prognostic significance of nuclear DNA content in mammary adenocarcinoma in humans. *Cancer Res.*, **44**, 394.

BLOOM, H.J. & RICHARDSON, W.W. (1957). Histological grading and prognosis in breast cancer. *Br. J. Cancer*, **11**, 359.

COULSON, P., THORNTHWAITE, J., WOOLLEY, T., SUGARBAKER, E. & SECKINGER, D. (1984). Prognostic indicators including DNA histogram type, receptor content, and staging related to human breast cancer patient survival. *Cancer Res.*, **44**, 4187.

DOWLE, C.S., OWAINATI, A., ROBINS, R.A. & 4 others (1987). The prognostic significance of the DNA content of human breast cancer. *Br. J. Surg.*, **74**, 133.

ELSTON, C., GRESHAM, G., RAO, G. & 4 others (1982). The Cancer Research Campaign (King's/Cambridge) trial for early breast cancer: clinico-pathological aspects. *Br. J. Cancer*, **45**, 655.

EWERS, S.B., LANGSTROM, E., BALDETROP, B., KILLANDER, D. (1984). Flow cytometric DNA analysis in primary breast carcinoma and clinicopathological correlations. *Cytometry*, **5**, 408

FOSSA, S., MARTON, P.F., KNUDSEN, O.S., KAALHUS, O., BORMER, O., VAAGE, S. (1982). Nuclear feulgen DNA content and nuclear size in human breast carcinoma. *Human Pathology*, **13**, 626.

HAYBITTLE, J.L., BLAMEY, R.W., ELSTON, C.W. & 5 others (1982). A prognostic index in primary breast cancer. *Br. J. Cancer*, **45**, 361.

HEDLEY, D., FRIEDLANDER, M., TAYLOR, I., RUGG, C., MUSGROVE, E. (1983). Method for analysis of cellular DNA content of paraffin-embedded pathological material using flow cytometry. *J. Histochem. Cytochem.*, **31**, 1333.

HEDLEY, D., RUGG, C., NG, A. & TAYLOR, I. (1984). Influence of cellular DNA content on disease free survival of stage II breast cancer patients. *Cancer Res.*, **44**, 5395.

HEDLEY, D., FRIEDLANDER, M. & TAYLOR, I. (1985). Application of DNA flow cytometry to paraffin embedded archival material for the study of aneuploidy and its clinical significance. *Cytometry*, **6**, 327.

HIDDEMANN, W., SCHUMANN, J., ANDREEFF, M. & 6 others (1984). Convention on nomenclature for DNA cytometry. *Cytometry*, **5**, 445.

NICHOLSON, R.I., CAMPBELL, F.C., BLAMEY, R.W. & 3 others (1981). Steroid receptors in early breast cancer: value in prognosis. *J. Steroid Biochem.*, **15**, 193.

OLSZEWSKI, W., DARZYNKIEWICZ, Z., ROSEN, P., SCHWARTZ, M. & MELAMED, M. (1981). Flow cytometry of breast carcinoma: 1. Relation of DNA ploidy level to histology and oestrogen receptor. *Cancer*, **48**, 980.

RABER, M.N., BARLOGIE, B., LATEREILLE, J., BEDROSSIAN, C., FRITSCHE, H. & BLUMENSCHEIN (1982). Ploidy, proliferative activity and ER content in human breast cancer. *Cytometry*, **3**, 36.

STUART-HARRIS, R., HEDLEY, D., TAYLOR, I., LEVENE, A. & SMITH, I. (1985). Tumour ploidy, response and survival in patients receiving endocrine therapy for advanced breast cancer. *Br. J. Cancer*, **51**, 573.

TAYLOR, I., MUSGROVE, E., FRIEDLANDER, M., FOO, M. & HEDLEY, D. (1983). The influence of Age on the DNA ploidy levels of breast tumours. *Eur. J. Cancer Clin. Oncol.*, **19**, 623.

THORUD, E., FOSSA, S.D., VAAGE, S. & 4 others (1986). Primary breast cancer: Flow cytometric DNA pattern in relation to clinical and histopathological characteristics. *Cancer*, **5**, 808.


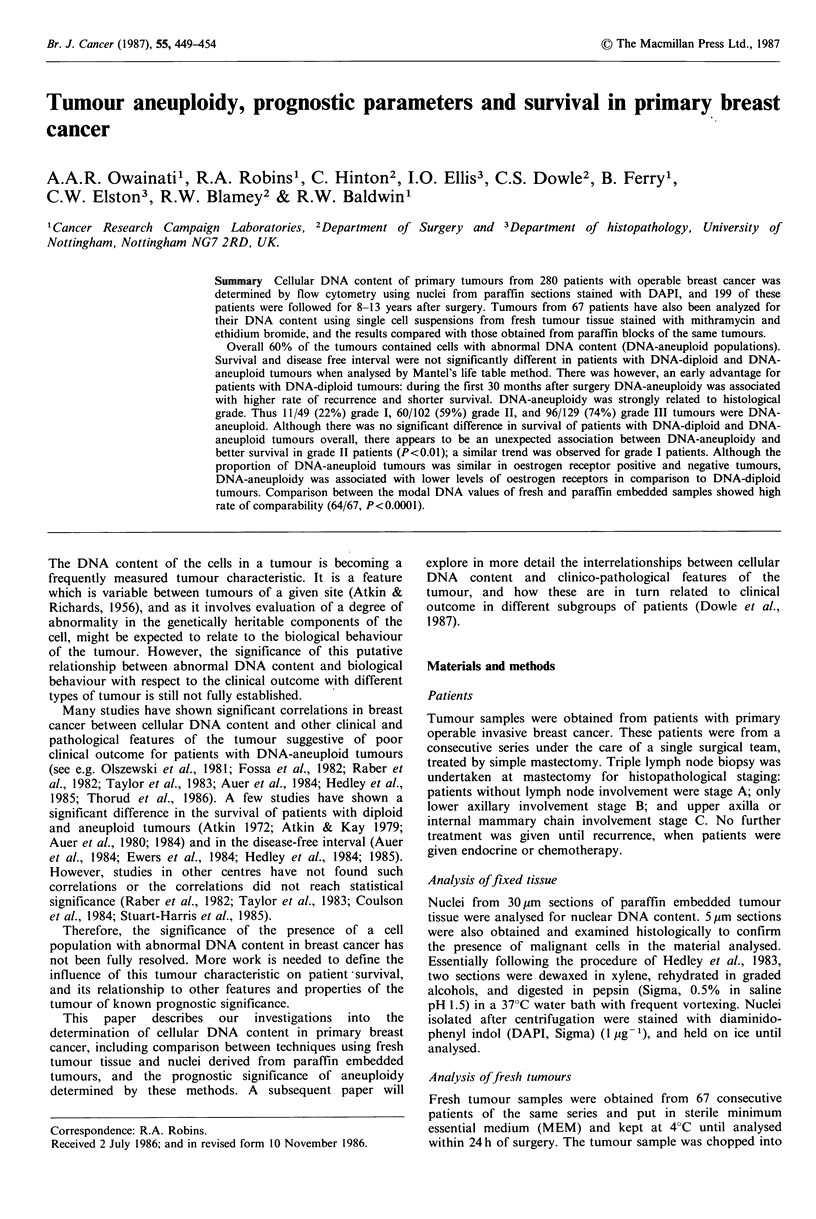

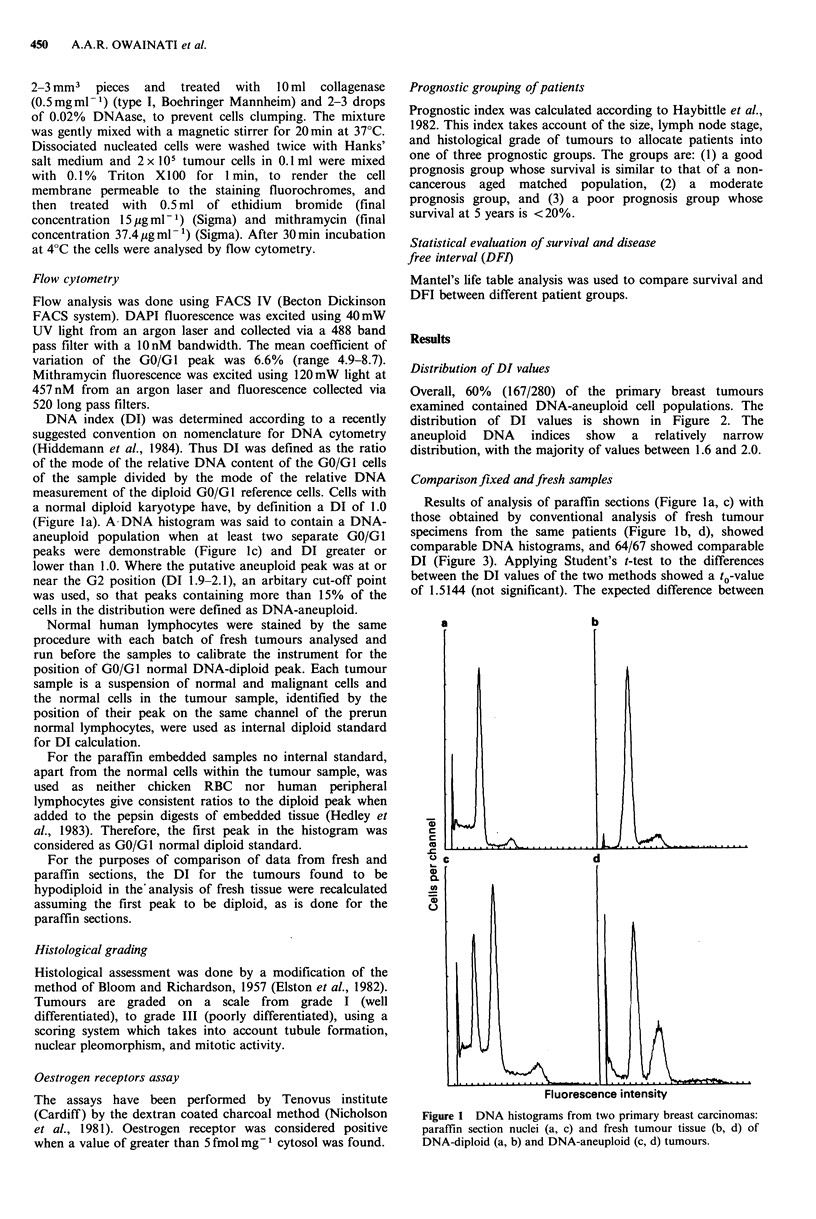

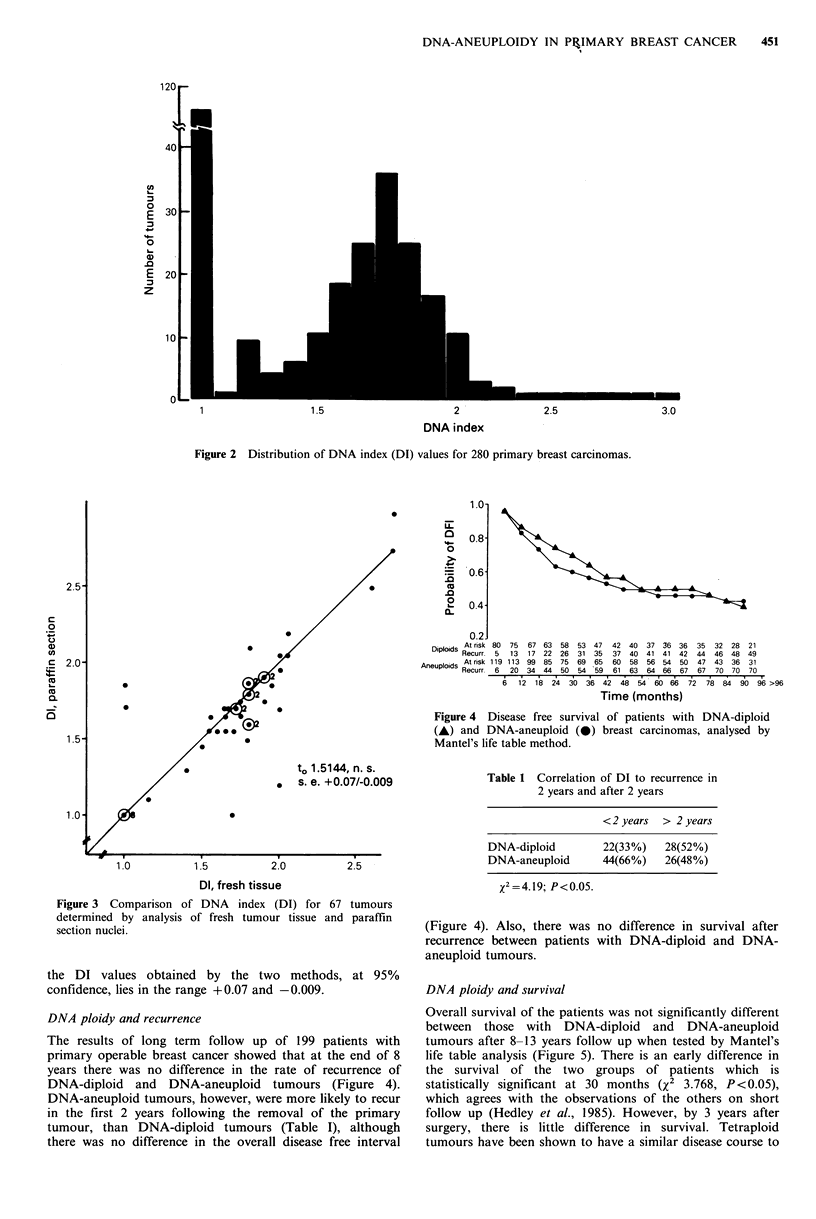

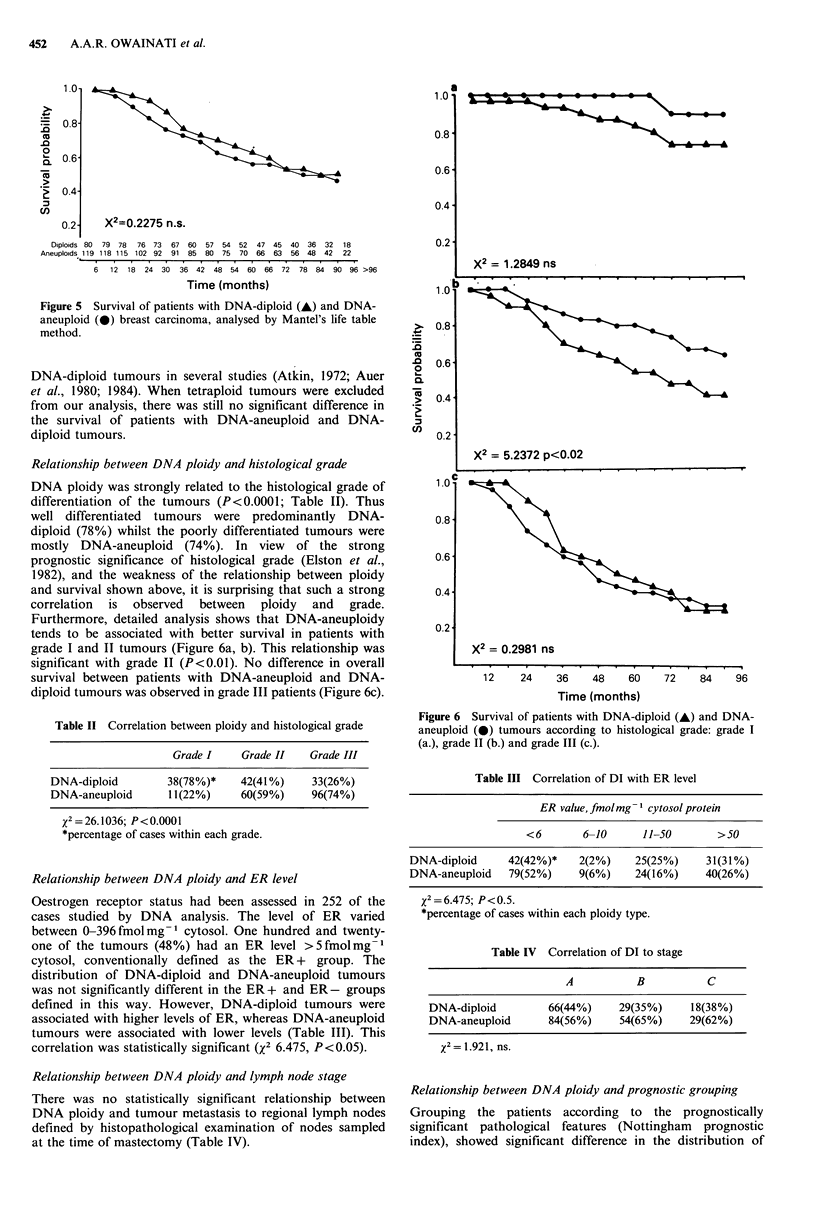

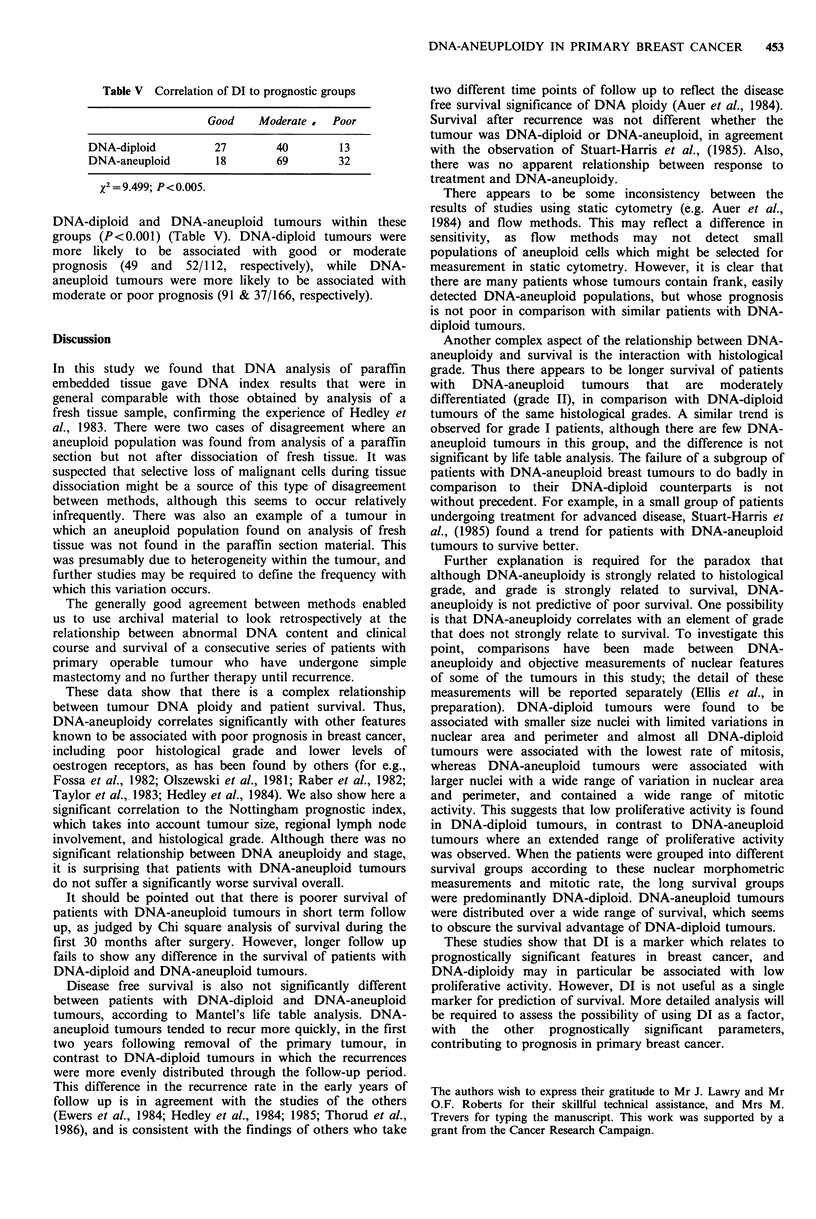

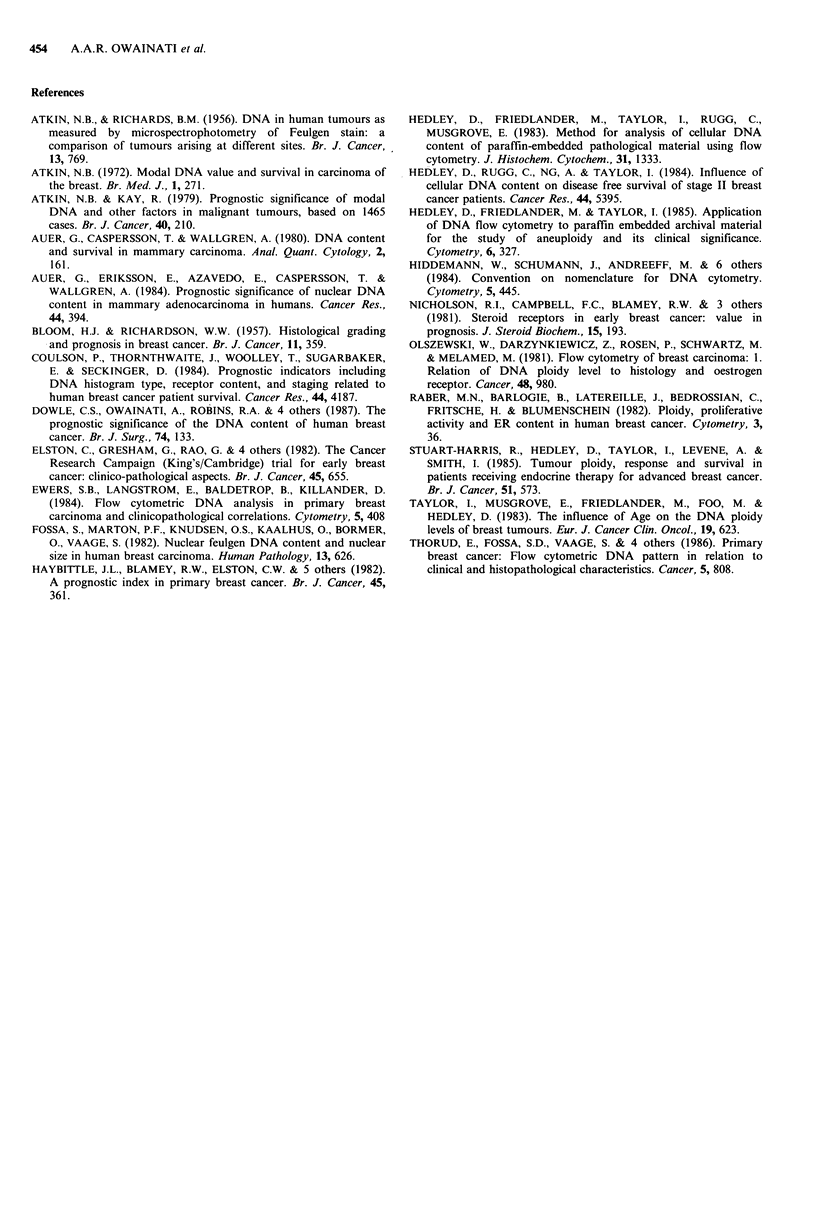


## References

[OCR_00727] ATKIN N. B., RICHARDS B. M. (1956). Deoxyribonucleic acid in human tumours as measured by microspectrophotometry of Feulgen stain: a comparison of tumours arising at different sites.. Br J Cancer.

[OCR_00737] Atkin N. B., Kay R. (1979). Prognostic significance of modal DNA value and other factors in malignant tumours, based on 1465 cases.. Br J Cancer.

[OCR_00733] Atkin N. B. (1972). Modal deoxyribonucleic acid value and survival in carcinoma of the breast.. Br Med J.

[OCR_00742] Auer G. U., Caspersson T. O., Wallgren A. S. (1980). DNA content and survival in mammary carcinoma.. Anal Quant Cytol.

[OCR_00747] Auer G., Eriksson E., Azavedo E., Caspersson T., Wallgren A. (1984). Prognostic significance of nuclear DNA content in mammary adenocarcinomas in humans.. Cancer Res.

[OCR_00753] BLOOM H. J., RICHARDSON W. W. (1957). Histological grading and prognosis in breast cancer; a study of 1409 cases of which 359 have been followed for 15 years.. Br J Cancer.

[OCR_00757] Coulson P. B., Thornthwaite J. T., Woolley T. W., Sugarbaker E. V., Seckinger D. (1984). Prognostic indicators including DNA histogram type, receptor content, and staging related to human breast cancer patient survival.. Cancer Res.

[OCR_00763] Dowle C. S., Owainati A., Robins A., Burns K., Ellis I. O., Elston C. W., Blamey R. W. (1987). Prognostic significance of the DNA content of human breast cancer.. Br J Surg.

[OCR_00768] Elston C. W., Gresham G. A., Rao G. S., Zebro T., Haybittle J. L., Houghton J., Kearney G. (1982). The cancer research campaign (King's/Cambridge trial for early breast cancer: clinico-pathological aspects.. Br J Cancer.

[OCR_00773] Ewers S. B., Långström E., Baldetorp B., Killander D. (1984). Flow-cytometric DNA analysis in primary breast carcinomas and clinicopathological correlations.. Cytometry.

[OCR_00777] Fosså S. D., Marton P. F., Knudsen O. S., Kaalhus O., Børmer O., Vaage S. (1982). Nuclear feulgen DNA content and nuclear size in human breast carcinoma.. Hum Pathol.

[OCR_00782] Haybittle J. L., Blamey R. W., Elston C. W., Johnson J., Doyle P. J., Campbell F. C., Nicholson R. I., Griffiths K. (1982). A prognostic index in primary breast cancer.. Br J Cancer.

[OCR_00798] Hedley D. W., Friedlander M. L., Taylor I. W. (1985). Application of DNA flow cytometry to paraffin-embedded archival material for the study of aneuploidy and its clinical significance.. Cytometry.

[OCR_00787] Hedley D. W., Friedlander M. L., Taylor I. W., Rugg C. A., Musgrove E. A. (1983). Method for analysis of cellular DNA content of paraffin-embedded pathological material using flow cytometry.. J Histochem Cytochem.

[OCR_00793] Hedley D. W., Rugg C. A., Ng A. B., Taylor I. W. (1984). Influence of cellular DNA content on disease-free survival of Stage II breast cancer patients.. Cancer Res.

[OCR_00809] Nicholson R. I., Campbell F. C., Blamey R. W., Elston C. W., George D., Griffiths K. (1981). Steroid receptors in early breast cancer: value in prognosis.. J Steroid Biochem.

[OCR_00814] Olszewski W., Darzynkiewicz Z., Rosen P. P., Schwartz M. K., Melamed M. R. (1981). Flow cytometry of breast carcinoma: I. Relation of DNA ploidy level to histology and estrogen receptor.. Cancer.

[OCR_00826] Stuart-Harris R., Hedley D. W., Taylor I. W., Levene A. L., Smith I. E. (1985). Tumour ploidy, response and survival in patients receiving endocrine therapy for advanced breast cancer.. Br J Cancer.

[OCR_00832] Taylor I. W., Musgrove E. A., Friedlander M. L., Foo M. S., Hedley D. W. (1983). The influence of age on the DNA ploidy levels of breast tumours.. Eur J Cancer Clin Oncol.

[OCR_00837] Thorud E., Fosså S. D., Vaage S., Kaalhus O., Knudsen O. S., Børmer O., Shoaib M. C. (1986). Primary breast cancer. Flow cytometric DNA pattern in relation to clinical and histopathologic characteristics.. Cancer.

